# Saccharated Calomel

**Published:** 1874-08

**Authors:** John M. Johnson

**Affiliations:** Atlanta, Ga.


### SACCHARATED CALOMEL.

By JOHN M. JOHNSON, M.D., Atlanta, Ga.

*Editor Atlanta Medical and Surgical Journal:*

Nearly a year ago an article of yours, in the Atlanta Medical and Surgical Journal, called attention to "saccharated" calomel, and, after some casting about, the credit of the discovery was accorded to me. I never claimed it for myself, but have often said I was the first man, and the only man, I had ever known to use it, except those who had done so under my advice. In 1871 or '72, while I was teaching Physiology in Atlanta Medical College, after delivering a lecture, in which I incidentally alluded to saccharated calomel, a young gentleman from Selma, Alabama, whose name I have forgotten, but who belonged to the class, told me that he was familiar with the preparation; but with this exception, I had never met a man who had used it, except as above stated.

It will scarcely be denied that to Hahnemann belongs the credit of attempting to utilize infinitesimals in the treatment of diseases. I do not know that he claimed in his day to have discovered and utilized saccharated calomel, not being very familiar with his books; but I do know that some of his followers claim it for him. I will not attempt to jostle his claim or that of anybody else to the honor of the discovery.

It is a cute little preparation, very convenient, very simple and very effective; and I stand ready to thank the gentleman, whoever he may turn out to be, who did first discover it. Among the objectors to the claim set up by my friends to originality in the matter is a gentleman who informs the public that the identical recipe is found in a small work on pharmacy, published in New York in 1858, by Dr. Horace Green, who commends very highly its cholagogue properties; another, that epilepsy was treated with calomel and sugar in 1804; and still another, that the giving of calomel on the tongue, with or without sugar, is a favorite method of exhibiting the remedy with several physicians whom he names. These gentlemen all speak truthfully, but they do not rise to the dignity of the question, which is not merely to combine calomel and sugar—that mode of treatment is perhaps as old as calomel itself; nor is it the giving of calomel, either combined or uncombined, loosely on the tongue, and mixing it with the saliva. The question is far greater, and rises immeasurably above all this. In a word, it means that by triturating half a grain of calomel, with two grains of hard loaf sugar, by a strong man, for fifteen minutes, that it will produce results that ounces were given to produce forty or fifty years ago.

One portion alone, given as hereafter directed, will often do all you want or expect; but if it does not, it will only be necessary to repeat it every two or three hours until it does.

Necessity is said to be the mother of invention, and man's progress in everything is due solely to his necessities. Nothing provokes exertion or enterprise like it. Nothing expands the mind and awakens the faculties so thoroughly. Who would study farming if the question of bread, and as a consequence ultimate existence itself, was not upon him? Who would study mechanics, often at the risk of life, and endure the sweat and dirt incident to such a life, if there was not a necessity and a reward? Who philosophy, if the present and future of events were settled? In a word, who would give all his powers to the science of discovery and utilization if it was not for the necessity which impels on the one hand, and the benefit accruing on the other?

In the early settlement of Kentucky there was developed a class of diseases fearful indeed in their malignity and fatality. Among these were "milk sickness," congestive fevers, bilious fevers, chills and fever, typhoid pneumonia, cold plague, as it was then called, or "head pleurisy," now known as cerebro-spinal meningitis, black tongue, malignant erysipelas, etc., etc. At that day medical culture was the exception, while empyricism and low quackery was the rule. Medical science was a sealed book; medical opinions undiffused and the most criminal, not to say infernal quackery, reigned every where, and reveled every where, unrebuked.

From 1825 to 1830, Transylvania University, at Lexington, began to be felt in all the departments of science and literature. A better class of men came upon the surface as teachers, lawyers, ministers and doctors; but unfortunately for the profession of medicine, the most heterodox theories, both as to the pathology and treatment of the diseases of the country, were taught. Dr. Jno. E. Cooke, Professor of the Theory and Practice of Medicine in the medical department of the University, taught the class that the true pathology of all the fevers of the country was the presence of black blood in the liver and vena cava, and the only port of safety was in large doses of calomel—twenty to fifty grains every two hours, until two or more ounces were administered, or until the black blood was purged out, or the patient died. Dr. B. W. Dudley, the friend of science, and the father of surgery in the West at that day, also lauded the value of the lancet with tartar emetic, and abstinence almost to starvation, as the great anchor of hope to the fevered man. Both of these were eminent men; both left the impress of their opinions broad and deep upon the susceptible minds of the students, who, on their part lost no time in returning home and putting into execution the heroic theories that had been so eloquently sounded in their ears during their pupilage. My father was a physician and a man of fine judgment, and with all a very conservative man. When my brother, Dr. Felix G. Johnson, long since dead, returned, after graduating, and gave the old man an account of what he had learned, he declared that it was all wrong; that an ounce of calomel was three times as much as a man ought to take, and, in fact, one hundred grains had been quite sufficient in his hands. Dr. Cook was the author of a pill which still bears his name, and is on sale in every drug store in the United States, I suppose. They were made with two grains each of colomel, aloes, and rhubarb. Of these I have seen a man—a lawyer of eminence—take, under the direction of two of the most eminent physicians of the town, seventy-five in one night to relieve milk sickness! and this with impunity.

The great question of the day was, can the patient be salivated? If so, he can get well; but if not, it is very doubtful. Everybody that recovered was salivated: Some with loss of teeth, not a few with sloughing of the lips and cheeks.

At this juncture Thompson, the father of the botanic system, made his advent. He gave the world his "complete system of medicine," in a book of less than one hundred pages. He boldly announced that he could cure anything in the nature of disease by his system, and the patient would never lose a meal. He denounced calomel, tartar emetic, blisters and the lancet, as the ministers of death and hell, and the public was warned from the pulpit and from the hustings to flee from this dread charnel-house to the city of refuge erected by the great reformer, Thompson. Nearly every man in the country bought a book and went to practicing the next day. Suits for mal-practice were talked about, and in some instances commenced. A vast majority refused to pay their bills, friends were estranged and open war raged everywhere. Legitimate medicine was tottering on its old foundation and reform must come. It did come. The heroic element gave place to moderate men, and Drake and Eberle, Yandell, Richardson and Short, and in the valley of the Mississippi, Cartwright, Meux, Luzenburg and Dowler. The microscope came, minute anatomy and histology began to unsettle the pompous theories of biggots. Physiology assumed new dignity and importance, and thus fell the altars and idols of an effete dynasty, and rational medicine began to take root, and while Thompsonianism nothing in itself, was the finger-board pointing to reform, the genius of rational induction has led the way to a glorious present, and let us hope, a still more glorious future.

It must be born in mind that at that day quinine was scarcely in use at all. Opium, as a means of relieving pain and equalizing the circulation, was taught theoretically, but few dared to use it beyond the smallest portions, whilst morphine was scarcely known even to the great body of practitioners. Calomel and tartar emetic were the Sampson and Herculean club of the materia medica; these, with blisters and the lancet, constituted the woof and webb of the stock in trade as taught and practiced by those to whom the profession looked for ideas.

In 1833, when less than twenty-one years old, I began the practice of legitimate medicine and surgery. I had never seen a medical college. But like Paul, after the strictest order of the sect, I lived a pharisee. My father and brother were both, in their day, eminent practitioners, my brother, a graduate of Transylvania. I was early indoctrinated into the ideas of the school, and lived strictly up to the faith delivered to me. Recognizing in calomel, tartar emetic, blisters and the lancet, remedies of great power and efficiency, and recognizing the further fact that some of them had been potent for mischief also, I set myself about turning the corners by which means I could achieve success and yet reveal nothing to the common mind which could in any way thwart the usefulness of my efforts. I substituted mustard, hot water, hot teas, scarification and cups for bleeding and blisters. I prepared, by rubbing them together, white powders of colomel and sugar, red powders of calomel, arminian bole and sugar, powders of calomel and prussian blue and sugar, and very often salts of nitre and calomel. These I put up in separate packages, and gave them in modified doses—first a red powder, then a blue, then a white, and then a strong powder, or nitre and calomel. These I placed on the tongue and washed them down with water.

None suspected they were taking calomel. As an after treatment in chills, I gave boneset in decoction and quinine in grain doses, every two or three hours. In continued fevers the after treatment was seneka tea, with camphor and quinine. In pneumonia I gave calomel and dover's powder, twenty grains each, mixed with sugar, and disguised with coloring matter. I gave this at two portions three hours apart. I applied hot blankets instead of blisters, and produced copious sweating instead of bleeding, and cured pneumonia then, as now, without the lancet.

About this time I formed a partnership with Dr. R. K. Linthicum, a man of ability and a student of Transylvania, now dead. He adhered to the doctrines into which he had been educated. He never compounded with friend or foe, but often rallied me severely about my quackery, as he called my little powders. We were partners seven years, thirty years ago. Our fortunes took diverging lines. He has passed away. I still live, at the age of sixty-three years, and am experimenting with my little powders and trying to develop everything that can be known of their mode of action, with the zeal of forty years ago.

In this crucible I learned the use of remedies, and here is the beginning of the use of saccharated calomel with me. From that day to this I have used it in preference to any other form. When I have simply desired to restore the secretions, and overcome costiveness, nothing ever yet discovered is comparable to it. In dysentery it is invaluable; also in diarrhœa, by combining the sixth of a grain of opium, its activity is restrained, but its effect on the secreting functions is always satisfactory.

In the costiveness of infants its action is everything that could be desired. In morning sickness, that greatest of trials alike to patients, doctors, and nurses, its influence is equally certain and happy. In all diseases where a limited, but certain effect, is desirable, it is just the remedy you want. In marasmus, anæmia, and consumption, where it is desirable to move the bowels, or to note the character of the secretions, without producing debility, nothing is so pleasant, so certain, so satisfactory.

But what is the rationale of its action? I hope I shall be able, before completing this paper, to settle this question forever. The reader will please recur to the circumstances narrated in the first part of this paper. It was absolutely necessary to take in sail, and scud before the storm, until its fury was exhausted. Our firm had a large country patronage, of plain people to be sure, but they were necessary to us. Concession had to be made to their opinions and prejudices. They believed that calomel was a destroying angel. They pointed to ruined health, disfigured faces and mouths, loss of teeth, etc., etc. It required great flexibility of mind to meet and turn aside these arguments. It was done however. I pledged myself that they should not be salivated unless to save their lives, and in that event I would tell them. I have heretofore given the formula of the different preparations of calomel, to-wit: calomel and sugar; calomel, sugar, and armenian bole; calomel, sugar, and prussian blue; and a strong powder of calomel and salts of nitre. There was in each five grains of calomel and sugar, and a moiety of coloring matter. I generally left one of each, amounting in all to twenty grains of calomel, which was a minimum portion at that time. These I ordered given at intervals of two to four hours—first a white, then a red, then a blue, and lastly a strong powder, placed on the tongue and washed down with a little water. To my astonishment I found that one powder almost invariably acted with such violence as to preclude any further exhibition of them. I then sub-divided the powders, and with nearly the same result; and I continued to do so until I had demonstrated the efficiency of half a grain. I commenced a system of experiments on myself. I took calomel in half grain doses, with and without sugar, with and without trituration, but had no result worth notice, excepting where the trituration had been premised. I observed, also, that if the stomach was not entirely empty the remedy had little or no effect; also, that if, when the stomach was empty, the dose was taken and kept in the mouth until a large amount of saliva had been collected, and then swallowed, the effect was invariable.

In giving this powder to infants, my rule has been to put it on the tongue, and then put the child to the breast, and no matter what has been the duration of the constipation, an action will follow in from two to four hours, without producing the least unpleasant effect whatever. For many years I believed that the lactic acid, produced by the sugar and milk in the stomach, induced the remarkable effects indicated. Another plausible theory was the conversion of the calomel into the bi-chloride of mercury, which, by concurring causes, was rendered soluble, and entered the circulation in this way.

In the course of a long observation of this remedy, I have marked it as a necessary concomitant to a satisfactory result that the stomach should be empty, and that it should be entangled in a large amount of saliva, or that it should be swallowed with new milk, whenever the mouth is too dry to furnish the necessary amount of saliva. Sugar and milk when taken into the stomach in limited quantities, are converted into lactic acid at once. The saliva having certain mixed properties, the most prominent of which is alkaline, and calomel being a chloride, the presence always of hydro-chloric acid in the stomach and lactic acid as an incident only, I believed that somewhere in this combination of elements, the transforming property existed which converted the calomel into a new agent, probably the bi-chloride of mercury. All this has been dispelled, however, and I am happy to be able to present a finality of its mode of action.

Several months ago I sought the assistance of my learned friend Dr. W. H. Goodwin, Professor of Chemistry in Atlanta Medical College, in assisting me to determine the effect of the trituration upon the calomel, and any other facts which might come to light bearing upon the subject. He demonstrates, conclusively that it is the mere comminution of the calomel molecules; being smaller than blood corpuscles they are capable of absorption and of transmission by the blood to the liver, and perhaps other secreting organs, and by its presence there promotes increased secretion.

I believe it is an accepted doctrine that constipation results from a want of normal secretion, not of bile alone, but of the pancreas and glands of the bowels as well, and perhaps of the spleen. This latter organ has the undoubted function of organizing products, but for what exact purpose is not apparent. The fact remains, however, that when the proper secretions are supplied, there is no constipation and no interruption of the healthy functions of the bowels.

If saccharated calomel obviates costiveness and restores a healthy condition of the bowels it must be by its action upon the whole series of secreting organs, and its mode of operation the transmission of the molecules to them and their presence there. I will now introduce Dr. Goodwin's letter:

January 4th, 1874.

Dr. Johnson:—On yesterday I triturated five grains of calomel with twenty of white sugar; then put three grains of the mixture in a test tube with an ounce of water. After shaking it well, and letting the sediment settle for ten minutes, I decanted the sediment from the liquid. The sediment reacted with lime water, ammonia and the iodide of potassium, to produce the mercuous oxide and iodide. The decanted liquid was then examined. It was semi-transparent, looking like a dilute solution of the acetate of lead. On being filtered, and the filter moistened with reagents, the presence of the murcuous chloride was manifest.

The filtered solution was made to change from its semi-transparent appearance to that of dilute ink, by the action of ammonia, though no precipitate was produced. I next made a simple solution of sugar in water; added calomel in the proportion of five grains of calomel to twenty grains of sugar; shook them up well with the water. After ten minutes the solution was perfectly transparent; the liquid, being decanted, gave no discoloration on the addition of aqua ammonia.

I then put three grains of the triturated article in an ounce of water, shaking them well together. After ten minutes the liquid was decanted from the sediment; the semi-transparent liquid was examined with an inch magnifying glass, and the cloudiness found to depend on the large number of small particles floating in it. On adding ammonia, the particles became dark and not so visible. I next examined both the semi-transparent liquid and that darkened by ammonia, with the compound microscope, with a half inch objective glass. The molecules in the liquids are about one-fifth the size of untriturated calomel, as shown by a comparison of the triturated and untriturated under the microscope. The molecule of the triturated calomel appears, under the microscope, when compared with a starch granule placed with it, about as much smaller than the starch granule as a common bird shot is smaller than a buckshot. The average size triturated particle is less than the human blood corpuscle. On yesterday, at 12 o'clock M., I took three grains of the triturated article, agitated it with an ounce of water, and decanted it after twenty minutes, from the sediment, and drank it. During the evening and last night I had pain and noise in the bowels. This morning, at light, I had a large, soft, and bilious evacuation.

You perceive, I only took what was floating in the liquid after the other portion of the three grains had settled. The sediment part is tinged a little yellow by trituration, as can be seen by comparison with moist untriturated calomel. Yet, chemically, it is mercurous chloride. I cannot account for the greater activity of the article on any other ground than its minute division.

Very respectfully, your friend,

W. H. Goodwin.

P. S.—I think all of the calomel in your prescription, by longer trituration, would be suspended in the sugar solution.

For more than thirty years I have known that saccharated calomel depended for its efficiency upon thorough trituration. During that time I have carefully observed and studied its therapeutical characteristics without being able to account for what I saw until the experiments of Prof. Goodwin revealed it to me. Since then I have given half grain of the medicine in a glass of champagne wine every two hours in morning sickness, with the happiest effects. I have given it in toast-water, gum water, sugar and water, tea and coffee, and the patient never suspected for a moment that he was taking calomel.

I submit this paper to the profession. I have written it because I was urged by many friends to do so. Nor do I expect to find myself famous on account of it. If light is thrown upon the subject I am content.

